# Influence of Match Status on Players’ Prominence and Teams’ Network Properties During 2018 FIFA World Cup

**DOI:** 10.3389/fpsyg.2019.00695

**Published:** 2019-03-28

**Authors:** Gibson Moreira Praça, Bernardo Barbosa Lima, Sarah da Glória Teles Bredt, Raphael Brito e Sousa, Filipe Manuel Clemente, André Gustavo Pereira de Andrade

**Affiliations:** ^1^Centro de Estudos em Cognição e Ação/ UFMG Soccer Science Center, Departamento de Esportes, Escola de Educação Física, Fisioterapia e Terapia Ocupacional, Universidade Federal de Minas Gerais, Belo Horizonte, Brazil; ^2^School of Sport and Leisure, Polytechnic Institute of Viana do Castelo, Viana do Castelo, Portugal; ^3^Instituto de Telecomunicações, Lisbon, Portugal

**Keywords:** football, Social Network Analysis, notational analysis, match status, playing position, situational variables, observational methodology

## Abstract

The analyses of players and teams’ behaviors during the FIFA World Cup may provide a better understanding on how football tactics and strategies have developed in the past few years in elite football. The Social Network Analysis (SNA) has been carried out in the investigations about passing distribution, improving the understanding on how players interact and cooperate during a match. In football official matches, studies have used the SNA as a means of coding players’ cooperation and opposition patterns. However, situational variables such as match status were previously investigated and associated with changes on teams’ dynamics within and/or between matches, but were not considered in studies based on Social Network Analysis. This study aimed to analyze the influence of match status on teams’ cooperation patterns and players’ prominence according to playing positions during 2018 FIFA World Cup. Fourteen matches of the knockout stage were analyzed. Macro and micro network measures were obtained from adjacency matrixes collected for each team, in each match status (winning, drawing, and losing). A one-way ANOVA was used to compare teams’ networks (macro-analysis variables) within each match status, while a two-way ANOVA (match status × playing position) was used to compare the micro-analysis variables. Results showed no differences between match status for macro analysis. Winning situations induced higher prominence in central midfielders (0.107; *p* = 0.001), wide midfielders (0.093; *p* = 0.001), and center forward (0.085; *p* = 0.001), while in losing situations lower prominence levels were observed for goalkeepers (0.044; *p* = 0.001) and center forward (0.074; *p* = 0.001). Data revealed that teams do not change macrostructures according to match status. On the other hand, the microstructures showed important adaptations regarding game styles, with changes in players’ behaviors according to playing positions. In general, the levels of centrality and prestige in players of different positions indicated a more direct play style in winning situations and a more build-up style in losing situations. These results allow a better understanding about the influence of match status on players’ and teams’ performance during high-level football competitions and may help coaches to improve athletes’ performance in these situations.

## Introduction

Notational match analysis provides valuable information about game dynamics (Hughes and Franks, 1997; Lago, 2009) and may help coaches to optimize training contents (Sarmento et al., 2015). Nevertheless, traditional notational analysis does not take into account the characteristics of the interactions between teammates (Clemente et al., 2015a). For this reason, the Social Network Analysis (SNA) has been also carried out in the investigations about passing distribution, improving the understanding on how players interact and cooperate during a match (Passos et al., 2011; Clemente et al., 2016). In football official matches, studies have used the SNA as a means of coding players’ cooperation and opposition patterns (Cotta et al., 2013; Gama et al., 2014; Clemente et al., 2015a). Macro and micro interaction patterns (i.e., interaction patterns related to the whole team or between individual players, respectively) may be analyzed through the passes performed between players and allow the understanding of the offensive phase dynamics. Typically, SNA applied to match analysis uses only the “last snapshot,” that is, the network resulting from the aggregate of all the interactions occurring during the entire match, focusing more on its structure and less on its dynamics (Ramos et al., 2018). However, situational variables such as match venue (García-Rubio et al., 2015), quality of the opposition (Taylor et al., 2008) and match status (Lago, 2009; Marcelino et al., 2011) were previously investigated and associated with changes on teams’ dynamics within and/or between matches. Therefore, the SNA may be performed during specific game phases, by including situational variables in the analysis, providing a broader and deeper understanding of players’ dynamics within a game.

In this context, match status is a situational variable characterized by a match momentary score (i.e., winning, drawing, or losing) and was previously reported to influence the offensive strategies within a football match (Taylor et al., 2008; Lago, 2009; Marcelino et al., 2011; Konefał et al., 2018). Previous studies showed that top European soccer teams preferred long passing sequences to achieve the opponent goal when they were losing or drawing and short passing sequences when they were winning (Paixão et al., 2015). Previous studies also demonstrated that losing teams tended to have greater ball possession (Lago and Martín, 2007). Besides, losing or drawing teams presented higher frequencies of passes, short passes, crosses, and longer ball possession time, leading to higher ball possession percentages (Konefał et al., 2018). Considering these results, we can expect losing teams to present a more cooperative behavior – characterized by higher values of density and clustering coefficient – in order to promote more imbalances on the defending team. However, to the best of our knowledge, no studies have previously examined the influence of match status on passing distribution with a SNA approach. The knowledge on how high-level football teams cope with losing or drawing match status may give insights to coaches on how to prepare their teams for these game situations.

In addition, players’ positional demands are an important aspect in football (Bradley et al., 2014). Considering the SNA approach, studies showed higher levels of prominence for midfielders (Clemente et al., 2015b), which are considered the key players for building the attack. However, it was also shown that situational variables, such as match location, can influence players’ positional demands. We expect, as previously reported, that losing teams may increase ball circulation to oppose a more defensive playing style preferred by the winning teams. This build up style, which is defined as long and controlled ball possessions in which a team is looking for opportunities to attack (Fernandez-Navarro et al., 2018) and captures teams’ strategy to safely progress into the field, could be perceived by an increased prominence of midfielders, since ball circulation is strongly based on their actions (Clemente et al., 2015b). On the other hand, a direct play style, which is characterized by instances of play where teams attempt to move the ball quickly toward the opposition’s goal through the use of long passes (Fernandez-Navarro et al., 2018) could be captured in winning teams by an expected higher frequency of links between distant players in the field [e.g., goalkeepers (GK) and forward]. However, these hypotheses were not tested in the literature.

The analyses of players’ and teams’ behaviors during the FIFA World Cup may provide a better understanding on how football tactics and strategies have developed in the past few years in elite football (Barreira et al., 2015). Besides, it has been shown that attacking teams had more difficulties to create favorable numerical contexts in the area of play than in the past (Barreira et al., 2015), what is expect to impact their possibility for passing networks and evidence the need of novel studies regarding this variable. In some recent events, the remarkable collective performance of Spain (2010) and Germany (2014) national teams (Cotta et al., 2013; Clemente et al., 2015a) – strongly based on positional game – has influenced many teams around the world and induced changes in the way that tactical principles are applied during the training process. Last year, France national team won the 2018 World Cup playing with a game style strongly based on defensive cohesion and fast offensive transitions, which is significantly different from the abovementioned teams. This fact demands an extensive incursion of scientists and coaches to understand what is new about football game principles and to comprehend the impact of this change in the “dominant style” for the next years. Based on this, the aim of this study was twofold: (1) to analyze the influence of match status on teams’ cooperation patterns and; (2) to analyze the influence of playing position on players’ prominence in different match status during the 2018 FIFA World Cup. We hypothesized that losing teams would present a higher cooperation between teammates and that losing situations would increase midfielders’ prominence.

## Materials and Methods

### Observational Design

According to the specific taxonomy of the area, the observational design was nomothetic, since we analyzed the behavior of multiple teams during the competition, i.e., there is a plurality of unities; followed-up intra-sessional and inter-sessional, because teams’ and players’ behaviors were analyzed throughout the match and throughout the knockout phase of the competition by a continuous recording with independent observation of each of the two opposing teams; and multidimensional, as multiple criteria (macro and micro levels of analysis) were taken into account (Anguera et al., 2011).

### Match Sample

We analyzed fourteen matches of the 2018 FIFA World Cup knockout stage. Two matches (Croatia 1 × 1 Denmark, and Belgium 2 × 0) were excluded because no significant time spent on different match statuses were observed. In these matches, more than 95% of the total time of the match spent in the same math status, which do not allow a better understanding about the influence of changing match status on teams and players’ behavior. The knockout phase comprised five stages: round of 16 (8 matches), quarter-finals (4 matches), semi-finals (2 matches), play-off for third place (one match), and final (one match). Within each match, there was one SNA adjacency matrix for each match status of each team. For example, during an Avs.B match, one matrix comprised the actions performed within the time interval during which team A was winning (e.g., 1 × 0). This matrix was accounted for the winning status (i.e., *n* = 1 for winning status). During this time interval, the actions of team B were comprised into another matrix, which was accounted for the losing status (*n* = 1 for losing status). In the case team B scored a goal (i.e., 1 × 1), the actions of each team were comprised into one matrix, both counted as drawing matrices (*n* = 2 for drawing status). A total of 58 adjacency matrices built based on unit of attacks were collected and treated. Therefore, our sample comprised 14 matches, with 58 changes in match status that resulted in 15 winning, 28 drawing, and 15 losing situations.

It is important to highlight that the present study of FIFA World Cup 2018 matches was not impacted by home-and-away effects because all the matches were played in Russia, eliminating or reducing the possible influence of game location. Following the qualifying tournament, only the 32 top worldwide national teams took part in the final stage, which reduced the impact of the competitive level.

This study was conducted in compliance with the Declaration of Helsinki.

### Data Collection and Analysis

Matches were monitored and recorded via the official broadcasting signal, publicly available. All matches were firstly split into three categories for each team: winning, losing, or drawing situations. Then, all matches were analyzed by two expert analysts, who counted all passes between teammates. Both analysts were tested for intra- and inter-reliability levels in a 21-day test-retest protocol using 14.28% of data as recommended in literature (Tabachnick and Fidell, 2007). Intraclass Correlation Coefficients (CCI 3,1) were calculated for both intra and inter-rater reliability, and the reliability levels were 0.997 for intra-observers and 0.980 for inter-observers, thus being considered enough for this type of observational analysis. In this study, passes were used to stablish the connections, as recommended in the literature (Clemente et al., 2015a). A successful pass occurred every time a player sent the ball to a teammate, who were able to keep the ball possession without any significant interference in ball trajectory by an opponent player.

**FIGURE 1:**
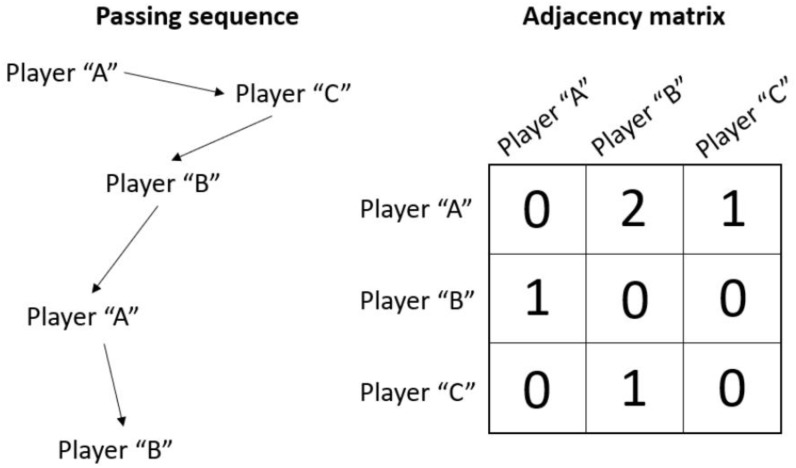
Example of a passing sequence and its respective adjacency matrix.

All analyses were carried out using the Ultimate Performance Analysis Tool – uPATO (Silva et al., 2019), which allowed the researchers to record information from the game, generate the adjacency matrixes, and analyze the measures of Social Network Analysis. Players were coded based on their position and only the playing position was considered for analyses not being necessary to standardize the time per each player. The weighted adjacency matrix was built based on the passing sequences (sequences of passes between teammates without interference of opponents or loss of the ball). Figure 1 provides an example of a passing sequence and its respective adjacency matrix. The final adjacency matrix of the match corresponds to the sum of all adjacency matrices built based on all the passing sequences performed by the team.

The general network properties of the SNA indicate the macro level of analysis, which are the interactions between players from a collective perspective (e.g., considering the whole team). The macro analysis includes the density and the clustering coefficient. The density is the ratio between the observed links (total links) and the maximum number of links (all possible links) (density values range from 0 – no density, lack of cooperation – to 1 – maximal cooperation). Links are the connections between two nodes (A to B). However, in the case of digraphs (our case), the links are considered in a bidirectional way, so that the connection can be stablished from player A to player B or from player B to player A depending on pass direction. The clustering coefficient indicates the level of interconnectivity between close teammates (values range from 0 – no density, lack of cooperation – to 1 – maximal cooperation) (Clemente et al., 2016).

The centrality measures are related to the level of prominence of a player in the game and indicates how effectively each player participated in the offensive process. In this study, three variables were analyzed: degree centrality (DC), degree prestige (DP), and page rank (PR). DC indicates the number of connections performed by a player. For this study, it indicates the percentage of passes performed by each player. Values range from 0 (lack of activity) to 1 (maximum exclusive centrality within the network). For instance, DP indicates the total number of connections received by a player. For this study indicates the percentage of passes received by a player. Values range from 0 (lack of activity) to 1 (maximum exclusive prestige within the network). PR indicates a player offensive popularity or the probability of a player to be activated. For this variable, values also range from 0 (lack of probability) and 1 (maximum exclusive popularity within the network).

**FIGURE 2:**
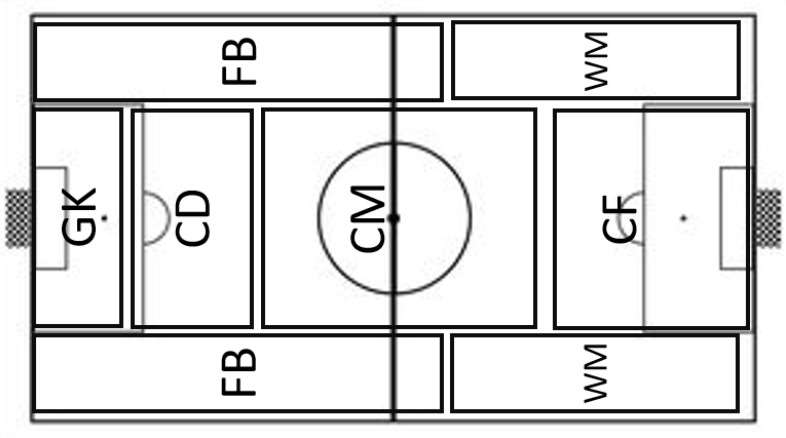
Classification of playing positions according to the area they mainly occupy in the field. GK, goalkeepers; FB, fullbacks; CD, central defenders; CM, central midfielders; WM, wide midfielders; CF, center forward.

For centrality measures, players were divided into six playing positions, as previously characterized in the literature: GK, fullbacks (FB), central defenders (CD), central midfielders (CM), wide midfielders (WM), and centre forward (CF) (Brito et al., 2017) (see Figure 2). The researchers defined players’ playing positions in all matches. When this classification was not consensual within the two analysts, playing positions were classified according to the players’ heatmap, available at https://www.fifa.com/worldcup/matches/. In these cases, the mean position occupied by the player in the field was used to define his playing position, according to the figure below:

### Statistical Analysis

Data were tested for normality (Shapiro–wilk’s test) and homocedasticity (Levene’s test). For macro analysis, a one-way ANOVA was used to compare data between the three different match status. In these analysis, $\eta_{p}^{2}$ effect sizes were calculated and classified in no effect ($\eta_{p}^{2}$ < 0.04), minimum effect (0.04 < $\eta_{p}^{2}$ < 0.25), moderate effect (0.25 < $\eta_{p}^{2}$ < 0.64), and strong effect ($\eta_{p}^{2}$ > 0.64) (Cohen, 1988). For micro analysis, a two-way ANOVA (match status × playing position) was used. In case of significant interactions, split analyses were performed within each factor. The r effect sizes were calculated (Field, 2009) for each significant pairwise comparison and classified as small effect (*r* = 0.10), medium effect (*r* = 0.30), and large effect (*r* = 0.50) (Cohen, 1988). In all cases, the level of significance was set at 5%. The statistical analyses were conducted in the statistical package SPSS (SPSS Version 19.0 for Windows, SPSS Inc., Chicago, IL, United States).

## Results

Table 1 presents the results of the comparison between the three different match status for macro analysis. No differences were reported for both variables (*p* > 0.05; no effect).

**Table 1:** Means (standard deviations) of the macro-analysis variables presented in each match status.

	Density	Clustering coefficient
Winning	0.481 (0.05)	0.167 (0.04)
Drawing	0.569 (0.03)	0.233 (0.031)
Losing	0.575 (0.04)	0.244 (0.042)
*p*-value	0.25	0.35
$\eta_{p}^{2}$	0.049	0.037


*$\eta_{p}^{2}$, effect size.*

There were significant interactions between factors (i.e., match status and playing positions) for DC, DP, and PR (*p* < 0.001). For this reason, pairwise comparisons were performed within each factor.

Table 2 presents the data (mean and standard deviation) for the degree centrality. The last column and the last line present the pairwise comparisons within each main factor. The comparison between match statuses within each playing position showed that GK (*r* = 0.286; small-to-medium effect) and CF (*r* = 0.320; medium-to-large effect) presented significantly higher values in winning situations compared to losing, while CD presented lower values in winning situations in comparison to drawing (*r* = 0.309 medium-to-large effect) and losing (*r* = 0.266; small-to-medium effect). Playing positions were also compared within each match status. In drawing situations, CD presented higher values than GK (*r* = 0.706; large effect), CM (*r* = 0.393 medium-to-large effect), WM (*r* = 0.529; large effect), and CF (*r* = 0.711; large effect). In the same match status, FB also presented higher values than CM (*r* = 0.577; large effect), CF (*r* = 0.572; large effect), and GK (*r* = 0.582; large effect).

**Table 2:** Means (standard deviations) of degree centrality presented by players of different playing positions according to match status.

Positions	Winning	Drawing	Losing	Pairwise comparisons – within each position
GK	0.078 (0.023)	0.044 (0.005)	0.040 (0.008)	W > L
FB	0.089 (0.009)	0.105 (0.007)	0.102 (0.008)	
CD	0.089 (0.010)	0.130 (0.006)	0.116 (0.007)	W < D,L
CM	0.108 (0.007)	0.096 (0.004)	0.112 (0.007)	
WM	0.086 (0.011)	0.076 (0.006)	0.081 (0.008)	
CF	0.075 (0.015)	0.050 (0.004)	0.043 (0.005)	W > L
Pairwise comparisons – within each match status		CD > GK,CM,WM,CF; FB > GK,WM,CF; WM > GK	GK,CF < FB,CD,CM,WM	


*GK, goalkeepers; FB, fullbacks; CD, central defenders; CM, central midfielders; WM, wide midfielders; CF, center forward; W, winning; D, drawing; L, losing. Pairwise comparisons indicate the significant differences within each main factor.*

Table 3 presents the data (mean and standard deviation) for the DP. The last column and the last line present the pairwise comparisons within each main factor Pairwise comparisons of match status within each playing position revealed a higher prestige for WM and CF in winning situations compared to drawing (*r* = 0.293; small-to-medium effect; and *r* = 0.298; small-to-medium effect) and losing (respectively: *r* = 0.209; small-to-medium effect; and *r* = 0.442; medium-to-large effect), and a lower prestige for CD in losing situations compared to winning (*r* = 0.526; large effect), and drawing (*r* = 0.193; small-to-medium effect). The comparisons between playing positions within each match status showed that, in winning situations, WM presented a higher prestige compared to GK (*r* = 0.489; medium-to-large effect), FB (*r* = 0,308; medium-to-large effect), and CD (*r* = 0.425; medium-to-large effect). In the same situation, CM and CF also presented higher values than GK (*r* = 0.478; medium-to-large effect compared with CM; and *r* = 0.574; large effect compared with CF) and CD (*r* = 0.321; medium-to-large effect compared with CM; and *r* = 0.420; medium-to-large effect compared with CF). In winning situations, GK also presented lower values than FB (*r* = 0.447; medium-to-large effect). In drawing situations, GK presented lower values than FB (*r* = 0.625; large effect), CD (*r* = 0.727; large effect), CM (*r* = 0.653; large effect), WM (*r* = 0.701; large effect) and CF (*r* = 0.607; large effect) while CD also presented a higher prestige than CF (*r* = 0.394; medium-to-large effect). In losing situations, GK and CF presented lower values than FB (respectively: *r* = 0.794; large effect; and *r* = 0.483; medium-to-large effect), CD (respectively: *r* = 0.684; large effect; and *r* = 0.436; medium-to-large effect), CM (respectively: *r* = 0.763; large effect; and *r* = 0.562; large effect), and WM (respectively: *r* = 0.805; large effect; and *r* = 0.568; large effect). These results indicated that forward players (CM, WM, and CF).

**Table 3:** Means (standard deviations) of degree prestige presented by players of different playing positions according to match status.

Positions	Winning	Drawing	Losing	Pairwise comparisons – within each position
GK	0.032 (0.007)	0.030 (0.005)	0.024 (0.004)	
FB	0.082 (0.011)	0.094 (0.006)	0.099 (0.008)	
CD	0.061 (0.008)	0.117 (0.006)	0.099 (0.008)	W < D,L
CM	0.103 (0.006)	0.095 (0.004)	0.110 (0.005)	
WM	0.141 (0.026)	0.092 (0.005)	0.104 (0.006)	W > D,L
CF	0.116 (0.017)	0.080 (0.006)	0.060 (0.006)	W > D,L
Pairwise comparisons – within each match status	WM > GK,FB,CD; CM,CF > GK,CD; FB > GK	GK < FB,CD,CM,WM,CF; CD > CF	FB,CD,CM,WM > GK,CF	


*GK, goalkeepers; FB, fullbacks; CD, central defenders; CM, central midfielders; WM, wide midfielders; CF, center forward; W, winning; D, drawing; L, losing. Pairwise comparisons indicate the significant differences within each main factor.*

Finally, Table 4 presents the data (mean and standard deviation) for the page rank. The last column and the last line present the pairwise comparisons within each main factor Pairwise comparisons within each position revealed that CD presented higher values in drawing situations compared to winning (*r* = 0.419; medium-to-large effect), and higher values for CF in drawing situations compared to losing (*r* = 0.347; medium-to-large effect). For comparisons between positions within match status, there was a higher prominence for CM in comparison to GK (*r* = 0.663; large effect), FB (*r* = 0.390; medium-to-large effect), CD (*r* = 0.541; large effect), and CF (*r* = 0.309; medium-to-large effect) in winning situations. WM also presented higher values than GK (*r* = 0.653; large effect) and CD (*r* = 0.396; medium-to-large effect) in winning situations. FB, CD, and CF presented higher values than GK (respectively: *r* = 0.600; large effect; *r* = 0.438; medium-to-large effect; and *r* = 0.598; large effect) in winning situations. In drawing situations, GK presented lower values than FB (*r* = 0.583; large effect), CD (*r* = 0.688; large effect), CM (*r* = 0.683; large effect), WM (*r* = 0.678; large effect), and CF (*r* = 0.702; large effect). Finally, in losing situations, CM and WM presented higher values than GK (respectively: *r* = 0.706; large effect; and *r* = 0.817; large effect), and CF (respectively: *r* = 0.427; medium-to-large effect; and *r* = 0.492; medium-to-large effect). GK also presented lower values than CF (*r* = 0.623; large effect).

**Table 4:** Means (standard deviations) of page rank presented by players of different playing positions according to match status.

Positions	Winning	Drawing	Losing	Pairwise comparisons – within each position
GK	0.045 (0.006)	0.046 (0.004)	0.044 (0.004)	
FB	0.081 (0.005)	0.088 (0.005)	0.086 (0.004)	
CD	0.070 (0.004)	0.093 (0.003)	0.083 (0.004)	W < D
CM	0.107 (0.005)	0.097 (0.003)	0.100 (0.004)	
WM	0.093 (0.007)	0.097 (0.004)	0.097 (0.004)	
CF	0.085 (0.007)	0.096 (0.005)	0.074 (0.005)	D > L
Pairwise comparisons – within each match status	CM > GK,FB,CD,CF; WM > GK, CD; FB,CD,CF > GK	GK < FB,CD,CM,WM,CF	CM,WM > GK,CF; CF > GK	


*GK, goalkeepers; FB, fullbacks; CD, central defenders; CM, central midfielders; WM, wide midfielders; CF, center forward; W, winning; D, drawing; L, losing. Pairwise comparisons indicate the significant differences within each main factor.*

## Discussion

This study aimed to analyze the influence of match status on teams’ cooperation patterns, as well as the influence of playing positions on players’ prominence in different match status during FIFA World Cup 2018. To the best of our knowledge, this is the first study to investigate the influence of situational variables, such as match status, on teams’ network properties and players’ prominence in SNA. Considering the well-stablished influence of situational variables on match dynamics, we believe this as an innovative contribution of the current study. Regarding the macro variables and the first aim of the study, no differences were observed between the different match statuses, probably because the absence of difference in both number and homogeneity of interactions. In this sense, differences only would be reported in macro variables if more (or more/less homogeneous) interactions were adopted by the teams, what was not observed. On the other hand, we found an increase in ball circulation in losing teams, corroborated by a higher prominence of midfielders and a lower prominence of GK and center forward, what indicates differences on game style. Therefore, micro variables can be affected by the differences in passing distribution (i.e., different players present higher prominence in different match statuses, although the team presents the same number of connections). Based on this, future studies should consider the concomitant analysis of macro and micro variables when trying to understand football.

The first aim of this study was to analyze the influence of match status on teams’ cooperation patterns during the 2018 FIFA World Cup. In general, center midfielders, wing midfielders, and center forward were the most prominent players in winning situations, which reinforces the assumption that this match status increased teams’ use of a direct play style. On the other hand, center forward were the lesser prominent in losing situations (except for GK), indicating a possible difficulty to achieve the most in-depth players when the teams were losing and demanding an increase in ball circulation to find good offensive opportunities. Clemente et al. (2015a) found higher values of density for winning teams during 2014 FIFA World Cup. Top teams also presented more ball touches, passes, and pass accuracy than bottom teams during the Spanish national championship “La Liga BBVA” 2012–2013, (Liu et al., 2015). Based on match outcomes, these results reinforce the idea that a more decentralized offensive process, based on passing progression and on a supporting progression strategy, could lead teams to be more successful during football matches. Additionally, a higher frequency of short passes was reported for winning teams in comparison to drawing and losing teams during the EURO 2016 (Konefał et al., 2018), and the total number of passes was positively associated with team performance (Pina et al., 2017). Based on these results, we hypothesized that winning teams would adopt a more defensive style in order to keep their favorable score. Therefore, losing teams would need a higher frequency of passes to build a better opportunity to find a scoring-box. Contrary to this hypothesis, we found no differences on both density and clustering coefficients between the three match statuses. Despite the higher frequency of passes found for winning teams reported in the literature (Liu et al., 2015), a previous study has demonstrated that the clustering coefficient is not a significant predictor of team performance, possibly indicating that different offensive styles can be equally effective for a team to succeed (Pina et al., 2017). Thus, this study results reinforce the rationale that multiple playing styles can be applied to achieve success in high-level football matches. Although in a macro level teams have presented a more stable dynamics, not influenced by match status (i.e., no differences for density and clustering coefficient), the existence of differences in micro variables suggests that the investigation of macro variables does not seem enough to capture changes in teams’ dynamics within a match, since no significant differences were detected.

The second aim of this study was to analyze players’ prominence according to playing positions in different match status during the 2018 FIFA World Cup. Since no interactions between main effects were observed, two different analysis were conducted for this aim: a comparison between different playing positions within each match status and a comparison between different match statuses within each playing position. In this topic, many studies have discussed the characteristics of each playing position during a football match (López-Peña and Touchette, 2012; Peixoto et al., 2017). For example, CM presented the highest frequencies of successful passes and receptions (Brito et al., 2017), and the highest prominence in official matches (López-Peña and Touchette, 2012; Peixoto et al., 2017). Considering the need to regain the ball possession quickly, losing teams are expected to adopt a more aggressive defensive style, increasing the time constraint to the team in offense, which is in line with the study of Barreira et al. (2015), who demonstrated that attacking teams increased their difficult to create a favorable numerical contexts in the game center over the years in modern soccer. By this, players in the offensive phase will be demanded to act in a time-constrained situation when winning, which will demand a faster process of gathering perceptual information and making the decisions, summed to a higher passing accuracy to overcome the opposing team. In this sense, midfielders can be more demanded during the game since recent studies showed that these players reached higher scores in positioning and deciding tactical skills (Kannekens et al., 2011) and a more position-specific coupling (Gonçalves et al., 2014). Therefore, the possible more aggressive defensive style observed in losing teams seems to increase the participation of midfielders as key players during this match status. Besides, this rationale may explain the lower prominence of these players during winning status. Finally, we can conclude that match status influences players’ prominence during official football matches.

Compared to winning teams, a previous study showed that losing teams decreased the direct play and maintenance play style, while increased the frequency of build up attacks (Fernandez-Navarro et al., 2018). The results of the present study reinforce these findings, which are in line with the changes in players’ prominence levels in the different match status. For example, a more direct play in winning situations may be inferred from the increased DP in CM, WM, and CF, the most advanced players on the pitch. These results indicate that a direct play strategy was possibly used by the winning teams to quickly build the attack and take advantage of the defensive derangements caused by the incomplete defensive transitions performed by the opposing team. On the other hand, losing situations were characterized by a reduced centrality and prestige for CF, indicating that these forward players were less activated in these situations. For these reasons, it is possible to infer that a ball circulation strategy (i.e., a build up style) was demanded, reducing the number of passes from and to CF. Therefore, in general, results showed that microstructures were sensitive to the influence of match status, while macrostructures were not.

This is the first study to examine the influence of match status on players’ and teams’ behavior using a SNA approach. The comprehension of the multi-faceted performance in football needs to be supported by techniques and tools suitable for capturing the dynamics of the game. However, results presented in the current study are limited to high-level football and not necessarily applicable to other contexts. Besides, changing in players’ positions during the matches were not considered in the present study, and should be addressed in future research. Future studies should also apply this novel approach in matches of different ages and competitive levels (regional, national, and international) to develop the road to expertise in youth academies.

## Conclusion

The network analysis of knockout phase of FIFA World Cup 2018 revealed that teams do not change macrostructures according to match status (i.e., losing, drawing, or winning). On the other hand, the microstructures showed important adaptations regarding game styles, with changes in players behaviors according to playing positions. In general, although the total of interactions stablished between players did not change significantly, the levels of centrality and prestige in players of different positions indicated a more direct play style in winning situations and a more build-up style in losing situations. These results allow a better understanding about the influence of match status on players’ and teams’ performance during high-level football competitions and may help coaches to improve athletes’ performance in these situations.

Coaches and analysts should analyze both macro and micro structures of a team to better comprehend its dynamics within and between matches and provide a quantitative feedback about the team and players performance. In this sense, a large behavioral variation within a match can be interpreted as a team’s difficulty to keep the game style during the match, indicating the need to improve players’ comprehension of team’s game principles and its application during higher pressure moments (i.e., losing situations). This issue is particularly important to national teams, because of their tight training schedule in comparison to lower level teams. On the other hand, during winning situations, it seems important to set up strategies for both direct play and ball maintenance to avoid a high frequency of long passes or unstructured progression in the field, which could decrease the offensive success and reduce the chances to keep the score. In both cases (winning and losing situations), training contents must contain the specific rules for progression or maintenance of ball possession, to allow players to develop their tactical knowledge of the team’s game principles needed during the matches.

## Data Availability

The datasets generated for this study are available on request to the corresponding author.

## Author Contributions

GP conceived the design, wrote the draft, and reached the final version. BL and RS collected the data, analyzed the matches, and provided the reviews to the draft. SB was responsible for the English version, supported the development of the research design, and wrote the draft. FC technically supported the procedures regarding match analysis, reviewed the draft, and analyzed the data. AA was responsible for the statistical procedures, interpretation of the data, and also reviewed the draft.

## Conflict of Interest Statement

The authors declare that the research was conducted in the absence of any commercial or financial relationships that could be construed as a potential conflict of interest.
